# The effect of supplementing with *Saccharomyces boulardii* on bismuth quadruple therapy for eradicating *Helicobacter pylori*: a systematic review and meta-analysis of randomized controlled trials

**DOI:** 10.3389/fmed.2024.1344702

**Published:** 2024-04-17

**Authors:** Yin Chen, Tao Teng, Yu Su, Wen-Zhong Chen

**Affiliations:** Department of Gastroenterology, Tongren People’s Hospital, Tongren, Guizhou Province, China

**Keywords:** *Saccharomyces boulardii*, *Helicobacter pylori*, probiotics, bismuth quadruple therapy, systematic review, meta-analysis

## Abstract

**Background and objective:**

It remains uncertain if the addition of *Saccharomyces boulardii* (*S. boulardii*) to bismuth quadruple therapy (BQT) recommended in the current guidelines can enhance the *Helicobacter pylori* (*H. pylori*) eradication rate and decrease the incidence of adverse events. We therefore conducted a meta-analysis of randomized controlled trials (RCTs) to address this issue.

**Methods:**

We performed comprehensive searches in PubMed, Embase, Web of Science, and Cochrane library databases from the inception of the databases through to November 1, 2023. A meta-analysis was conducted to determine the pooled relative risk (RR) with 95% confidence intervals (CI) using a random-effects model. We utilized the revised Cochrane Risk of Bias Tool to assess the risk of bias of included studies.

**Results:**

A total of six RCTs (1,404 patients) included in this meta-analysis. The results of the intention-to-treat analysis showed that the combination of *S. boulardii* with BQT had a higher eradication rate than BQT alone (87.0% versus 83.3%), with a pooled RR of 1.05 (95% CI: 1.00–1.10, *p* = 0.03). In the per-protocol analysis, however, there was no statistical significance between the two groups in the eradication rate (93.7% versus 91.0%, RR = 1.03, 95% CI: 1.00–1.06, *p* = 0.07). The combination of *S. boulardii* and BQT had a significantly lower rate of overall adverse events (22% vs. 39%, RR = 0.56, 95% CI: 0.44–0.70, *p* < 0.00001), diarrhea (7.9% vs. 25.7%, RR = 0.29, 95% CI: 0.17–0.48, *p* < 0.00001), constipation (2.9% vs. 8.4%, RR = 0.35, 95% CI: 0.14–0.88, *p* = 0.03) and abdominal distention (4.9% vs. 12.7%, RR = 0.41, 95% CI: 0.23–0.72, *p* = 0.002) than BQT alone. For the assessment of risk of bias, five studies were deemed to have some concerns, while one study was judged to have a low risk.

**Conclusion:**

Current evidence suggests that supplementation with *S. boulardii* in BQT may not have a major effect on the *H. pylori* eradication rate, but significantly reduces the incidence of overall adverse events, diarrhea, abdominal distention and constipation. Combining *S. Boulardii* with BQT can help alleviate symptoms, potentially improving patient adherence.

**Systematic review registration:**

https://osf.io/n9z7c.

## 1 Introduction

*Helicobacter pylori* (*H. pylori*) is a Gram negative, spiral-shaped microbe that colonizes the stomach and has become a major public health concern, with more than half of the world’s population affected by it (1, 2). It is a widely accepted notion that *H. pylori* infection is correlated with a range of gastrointestinal ailments, including chronic gastritis, peptic ulcer disease, gastric mucosa-associated lymphoid tissue (MALT) lymphoma, and gastric cancer. In the past few years, numerous studies have revealed that *H. pylori* infection is not only the cause of gastrointestinal issues, but could also be associated with a variety of extragastrointestinal illnesses including cardiovascular, hematological, neurological, metabolic, and skin diseases (3–5). By eliminating *H. pylori* in its initial stages, the chances of developing gastric cancer can be significantly reduced (6, 7).

In the past, standard triple therapy (STT), which was made up of a proton pump inhibitor (PPI) and two antibiotics (amoxicillin and clarithromycin/metronidazole), was the most common approach to eradicating *H. pylori*. However, the growing prevalence of antibiotic resistance has complicated attempts to eradicate *H. pylori*, particularly with regards to clarithromycin resistance, and thus clarithromycin triple therapy may no longer be the most suitable first-line treatment (3, 8). Currently, the bismuth quadruple therapy (BQT) for 10–14 days is first-line treatment that are recommended in several guidelines and consensus reports (3, 9–11). Although BQT is an effective method for eradicating *H. pylori* infection, adverse events and poor compliance during the eradication process are common (12, 13). Additionally, increasing evidence points to the fact that eradication drugs, especially antibiotics and PPI, can cause an imbalance in the gut microbiota, which has a significant impact on human health (14, 15). Therefore, new therapies are needed.

Probiotics, as living microorganisms, have been employed extensively to treat illnesses like antibiotic-induced diarrhea, colitis, and metabolic syndrome (16). *Saccharomyces boulardii* (*S. boulardii*) is the only probiotic preparation derived from fungi that is used worldwide. A meta-analysis by Szajewska et al. (17) demonstrated that the supplementation of *S. boulardii* in the STT for *H. pylori* can result in a higher eradication rate and a lower incidence of side effects. The resistance to antibiotics has increased significantly, making STT no longer as effective as before. It remains uncertain if the addition of *S. boulardii* to BQT recommended in the current guidelines can enhance the *H. pylori* eradication rate and decrease the incidence of adverse events. Several randomized controlled trials (RCTs) have been conducted on this topic in recent years, yet the small sample size of each study has not allowed for any definite conclusions to be made. To evaluate the efficacy and safety of *S. boulardii* assisted BQT versus BQT, we therefore performed this systematic review and meta-analysis.

## 2 Materials and methods

This systematic review was conducted in accordance with the PRISMA 2020 statement (18). The protocol of this study was registered in the Open Science Framework.^1^

### 2.1 Literature search

Utilizing pre-determined search terms, we performed systematic searches in PubMed, Embase, Web of Science, and the Cochrane library database, up to November 1, 2023, without any language limitations. The search terms included “*helicobacter pylori*,” “*H. pylori*,” *helicobacter*, “*campylobacter pylori*,” “saccharomyces boulardii,” “*S. boulardii*,” probiotics, probiotic, “bismuth.” Taking PubMed as an example, the detailed search strategy was follow: (“*helicobacter*”[MeSH Terms] OR “*helicobacter*”[tiab] OR “*helicobacter pylori*”[MeSH Terms] OR “*helicobacter pylori*”[tiab] OR “*H. pylori*”[tiab] OR “*campylobacter pylori*”[tiab]) AND (“*saccharomyces boulardii*”[tiab] OR “*S. boulardii*”[tiab] OR probiotics[MeSH] OR probiotics[tiab] OR probiotic[tiab]) AND (“bismuth”[MeSH Terms] OR “bismuth”[tiab]). The retrieval strategies for the other three electronic databases are detailed in Supplementary Table S1. Additionally, we examined the reference lists of the evaluated studies to identify any further eligible studies.

### 2.2 Study selection

Two reviewers (Chen Y and Teng T) independently conducted two screenings of the study selection. They initially evaluated the title and abstract of the articles and excluded those that were unlikely to be related to the research. Then, the two reviewers examined the full-text articles and chose those that were eligible for meta-analysis.

Studies that fulfilled the following criteria were included: (1) Study design: RCTs; (2) Participants: adults patients who have not had any professional treatment for *H. pylori* in the past; (3) Intervention: Combined treatment of *S. boulardii* with BQT; (4) Comparison: the same BQT (bismuth + PPI + two antibiotics); and (5) Outcomes: *H. pylori* eradication rate and the incidence of adverse events (including the overall and specific adverse events). We will not consider studies on other probiotics, duplicates, Non-RCTs, animal experiments, reviews and meta-analysis, conference abstracts, letters, editorials, guidelines and consensus, and studies from which data cannot be gathered.

### 2.3 Data extraction and risk of bias

The data collected from the eligible studies included the name of the first author, year of publication, country of origin, sample size, diagnosis methods used for *H. pylori*, information regarding the intervention and control groups, and relevant data on the outcomes of interest.

We utilized the revised Cochrane Risk of Bias Tool (RoB 2.0) for randomized trials to assess the risk of bias in the included studies, encompassing the following five domains: (1) bias arising during randomization; (2) bias due to deviations from intended interventions; (3) bias from missing outcome data; (4) bias in outcome measurement; and (5) bias in reporting outcome selection. The bias risk in each category can be categorized into three levels: low risk of bias, some concerns, and high risk of bias (19). Two reviewers (Chen Y and Teng T) independently collected data and assessed the risk of bias for each study, and any disagreements were resolved through consensus.

### 2.4 Statistical analysis

The relative risk (RR) and 95% confidence intervals (CIs) were calculated as summary effect size following the random-effects model. To assess the heterogeneity between studies, both the I^2^ statistic and the chi-square test with a *p* value <0.10 were employed. If the *p* value was <0.10, substantial heterogeneity was determined. Heterogeneity was categorized as insignificant, low, moderate, or high, depending on the I^2^ values, which were 0–25%, 26–50%, 51–75%, and above 75%, respectively (20). Data for *H. pylori* eradication rate were analyzed using both intention-to-treat (ITT) and per-protocol (PP) analysis. The ITT analysis involved all participants who were initially assigned to the group through random selection. The PP analysis excluded patients who did not withdraw for any reason and received treatment doses below 90%. We performed pre-specified subgroup analyses by duration of BQT, dosage of *S. boulardii* and duration of supplementation with *S. boulardii*. Subgroup analyses of *H. pylori* eradication rate were conducted through ITT analysis. The publication bias should be investigated by funnel plot and Egger test if at least 10 studies are included in the meta-analysis (21, 22). It was determined that a *p*-value of less than 0.05 was indicative of a significant publication bias. All analyses were conducted using the RevMan 5.3 software (the Cochrane Collaboration, Copenhagen, Denmark) and STATA/SE (Version 12.0, STATA Corporation, Texas, United States).

## 3 Results

### 3.1 Study selection

**Figure 1:**
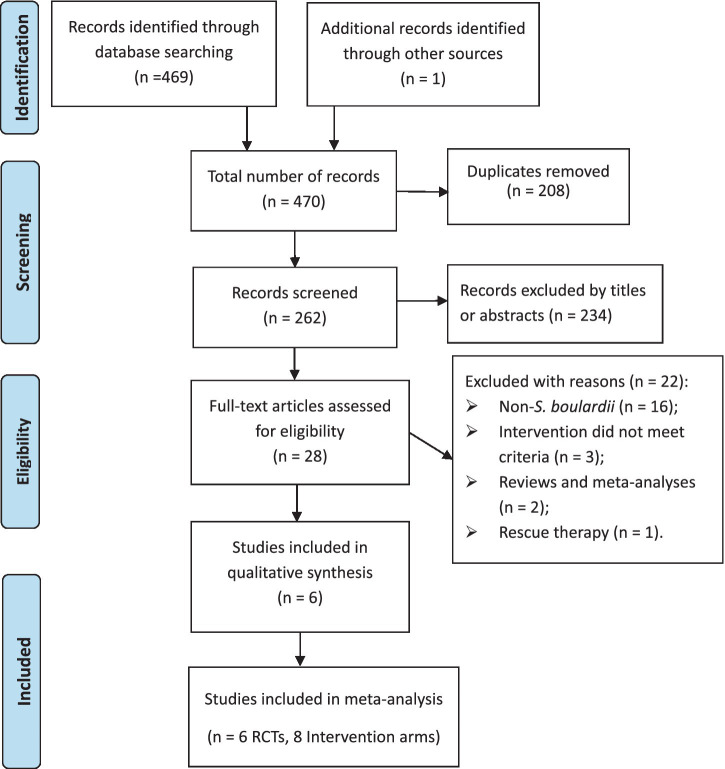
PRISMA flowchart of study selection process.

System retrieval produced 470 records, of which 208 were duplicates, leaving 262 records. After screening titles and abstracts, 234 records were excluded, leaving 28 full-text articles to be reviewed. Ultimately, 6 RCTs (8 intervention arms) (23–28) were included in the meta-analysis, as illustrated in Figure 1.

### 3.2 Study characteristics

A total of six RCTs (involving 1,404 patients) published from 2017 to 2023 included in our meta-analysis. All studies originate from Asia, five of which are from China (23–26, 28) and one from Iran (27). Of all the studies, one was a multicenter RCT (26), while the remaining five were single center RCTs. Two RCTs (23, 25) included multiple arms. The number of participants in these RCTs varied from 104 to 348. Regarding the duration of treatment for BQT, there were three studies (23–25) with a duration of 10 days and the other three studies (26–28) with a duration of 14 days. The dosage of *S. boulardii* varied between studies, with one study (27) using 500 mg/day and the others 1,000 mg/day (23–26, 28). The duration of *S. boulardii* regimen was 14 or 28 days in one study, while the other five studies only had 14 days of treatment. The major characteristics of the studies incorporated are outlined in Table 1.

**Table 1:** Main characteristics of included studies.

Study	Country	Study design	No. of patients (*S. boulardii* + BQT/BQT)	BQT regimen	*S. boulardii* regimen (dosage, duration)	Diagnositic methods of *H. pylori*	
						Initial	Rechecking
Zhu 2017 (23)	China	Single-center RCT	240 (80/80/80)	Bismuth potassium citrate 220 mg bid, rabeprazole 10 mg bid, amoxicillin 1,000 mg bid, furazolidone 100 mg bid, 10 days	500 mg bid, Group I: 14 days; Group II: 28 days	UBT	UBT
Zhu 2018 (24)	China	Single-center RCT	240 (120/120)	Bismuth potassium citrate 220 mg bid, esomeprazole 20 mg bid, amoxicillin 1,000 mg bid, furazolidone 100 mg bid, 10 days	500 mg bid, 14 days	UBT	UBT
He 2019 (25)	China	Single-center RCT	300 (100/100/100)	Bismuth potassium citrate 220 mg bid, pantoprazole 40 mg bid, amoxicillin 1,000 mg bid, furazolidone 100 mg bid, 10 days	500 mg bid, 14 days Group I: simultaneous administration; Group II: administration after BQT	UBT	UBT
Zhao 2021 (26)	China	Multicenter RCT	348 (169/179)	Bismuth potassium citrate 220 mg bid, esomeprazole 20 mg bid, amoxicillin 1,000 mg bid, clarithromycin 500 mg bid, 14 days	500 mg bid, 14 days	UBT	UBT
Naghibzadeh 2022 (27)	Iran	Single-center RCT	104 (52/52)	Bismuth subcitrate 120 mg bid, esomeprazole 40 mg bid, amoxicillin 1,000 mg bid, clarithromycin 500 mg bid, 14 days	250 mg bid, 14 days	Histology	SAT
He 2023 (28)	China	Single-center RCT	172 (86/86)	Colloidal bismuth pectin 200 mg tid, Esomeprazole 20 mg bid, antofloxacin 200 mg qid, amoxicillin 1,000 mg bid, 14 days	500 mg bid, 14 days	UBT or histology	UBT

BQT, bismuth quadruple therapy; RCT, randomized controlled trial; bid, twice daily; tid, three times daily; qid, four times daily; UBT, urea breath test; RUT, rapid urease test; SAT, stool antigen test.

### 3.3 Risk of bias

All of the six RCTs included in the analysis exhibited a low risk of bias in terms of the randomization process, missing outcome data, measurement of the outcome, and selection of reported results. In relation to bias from deviations in the intended intervention, five studies were deemed to have some concerns, while one study was judged to have a low risk. The details of the risk of bias are presented in Supplementary Figures S1, S2.

### 3.4 *Helicobacter pylori* eradication rate

**Figure 2:**
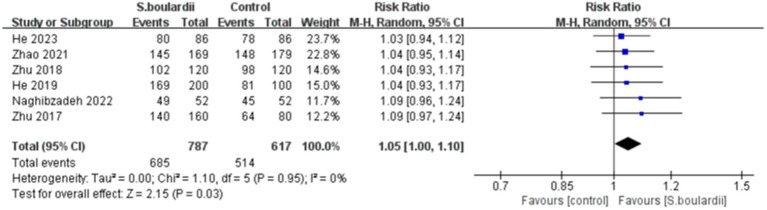
Forest plot of the *H. pylori* eradication rate (ITT data).

**Figure 3:**
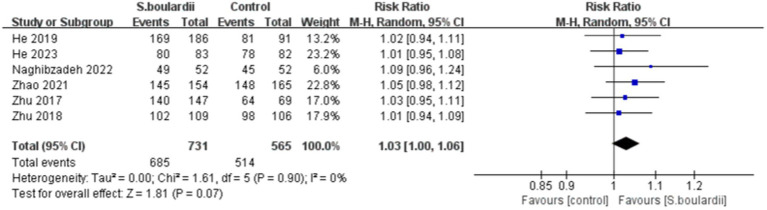
Forest plot of the *H. pylori* eradication rate (PP data).

Six RCTs with 1,404 participants reported data on the *H. pylori* eradication rate. The results of the ITT analysis showed that the combination of *S. boulardii* with BQT had a higher eradication rate than BQT alone (87.0% versus 83.3%), with a pooled RR of 1.05 (95% CI: 1.00–1.10, *p* = 0.03) and no heterogeneity (I^2^ = 0%, *p* = 0.95) (Figure 2). In the PP analysis, the eradication rate of *S. boulardii* in combination with BQT was higher than BQT alone (93.7% versus 91.0%), however, there was no statistical significance between the two groups (RR = 1.03, 95% CI: 1.00–1.06, *p* = 0.07) (Figure 3). No statistical heterogeneity was observed (I^2^ = 0%, *p* = 0.90).

Based on ITT analysis data, we further conducted subgroup analyses based on duration of BQT regimen, dosages of *S. boulardii*, and duration of *S. boulardii* to explore the potential influencing factor on the overall results. Results from the subgroup analysis based on duration of BQT regimen showed that the *H. pylori* eradication rate was higher in the *S. boulardii* supplementation group in the 10-day subgroup (*n* = 3 RCTs, RR = 1.06, 95% CI: 0.99–1.13, *p* = 0.11) and 14-day subgroup (*n* = 3 RCTs, RR = 1.04, 95% CI: 0.99–1.10, *p* = 0.15), yet the difference was not statistically significant. Results from the subgroup analysis based on dosages of *S. boulardii* showed that the *H. pylori* eradication rate was higher in the *S. boulardii* supplementation group in the subgroup of 500 mg/day (*n* = 1 RCT, RR = 1.09, 95% CI: 0.96–1.24, *p* = 0.19) and the subgroup of 1,000 mg/day (*n* = 5 RCTs, RR = 1.04, 95% CI: 1.00–1.09, *p* = 0.07), yet the difference was not statistically significant. In a subgroup analysis based on duration of *S. boulardii*, the *H. pylori* eradication rate increased significantly in the 14-day subgroup (*n* = 6 RCTs, RR = 1.05, 95% CI: 1.00–1.09, *p* = 0.04), but not in the 28-day subgroup (*n* = 1 RCT, RR = 1.09, 95% CI: 0.95–1.25, *p* = 0.20). The results of subgroup analyses were summarized in Table 2.

**Table 2:** Results of subgroup analyses of *H. pylori* eradication rate (ITT data).

Subgroups	No. of studies	Sample size	RR (95%CI)	*P*_effect_	I^2^ (%)	*P*_heterogeneity_
Duration of BQT regimen						
10 days	3	780	1.06 (0.99–1.13)	0.11	0	0.81
14 days	3	624	1.04 (0.99–1.10)	0.15	0	0.74
Dosages of *S. boulardii*						
500 mg /day	1	104	1.09 (0.96–1.24)	0.19	–	–
1,000 mg /day	5	1,300	1.04 (1.00–1.09)	0.07	0	0.95
Duration of *S. boulardii*						
14 days	6	1,324	1.05 (1.00–1.09)	0.04	0	0.96
28 days	1	160	1.09 (0.95–1.25)	0.20	–	–

ITT, intention-to-treat; RR, relative risk; CI, confidence interval; RCTs, randomized controlled trials.

### 3.5 Adverse events

**Figure 4:**
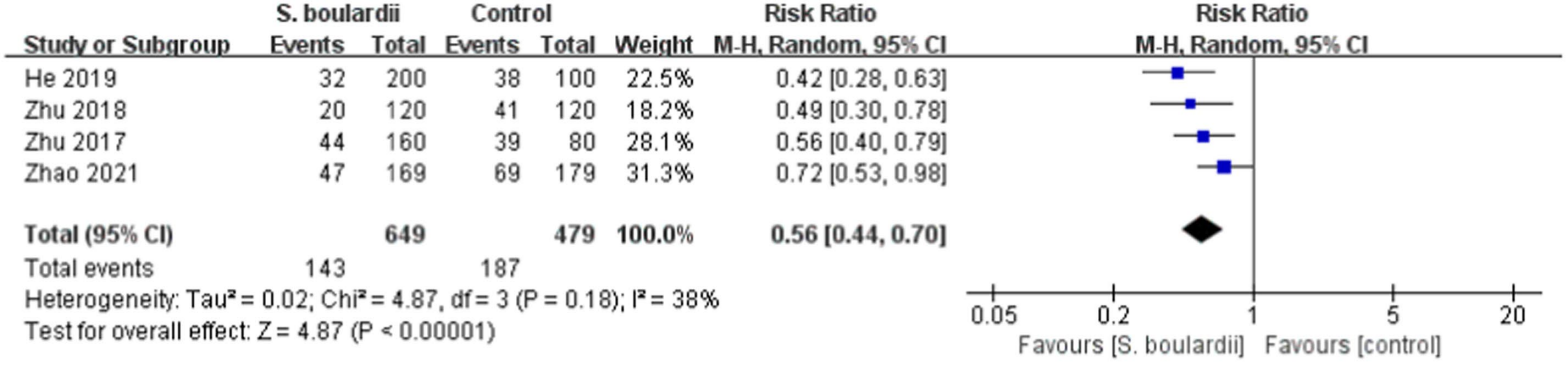
Forest plot of the overall adverse events.

Four RCTs (23–26) involving a total of 1,128 participants revealed the incidence of overall adverse events. Results of the meta-analysis showed that the combination of *S. boulardii* and BQT had a significantly lower rate of overall adverse events than BQT alone (22% vs. 39%, RR = 0.56, 95% CI: 0.44–0.70, *p* < 0.00001). The heterogeneity was low (I^2^ = 38, *p* = 0.18) (Figure 4).

**Figure 5:**
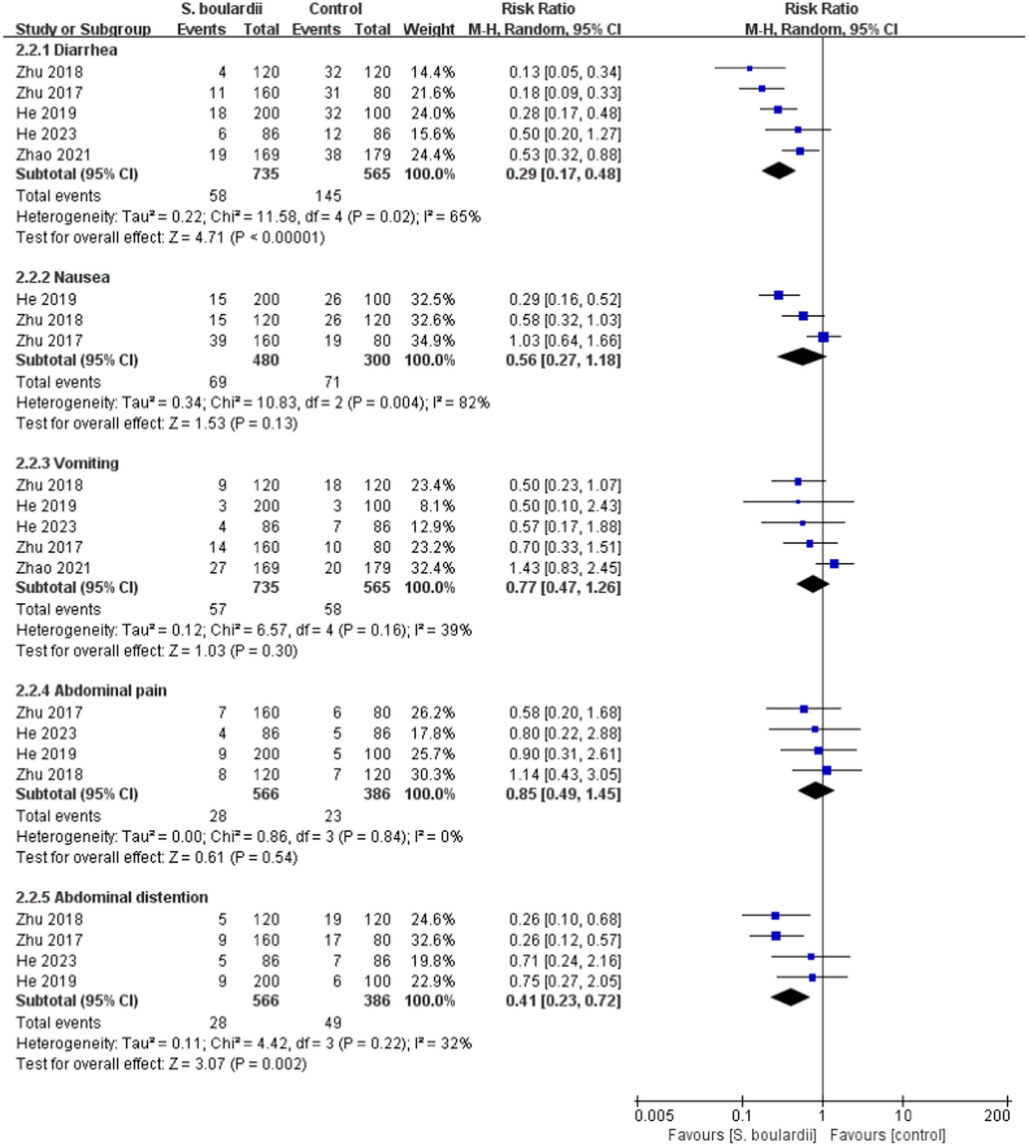
Forest plot of the specific adverse events (diarrhea, nausea, vomiting, abdominal pain, and abdominal distention).

**Figure 6:**
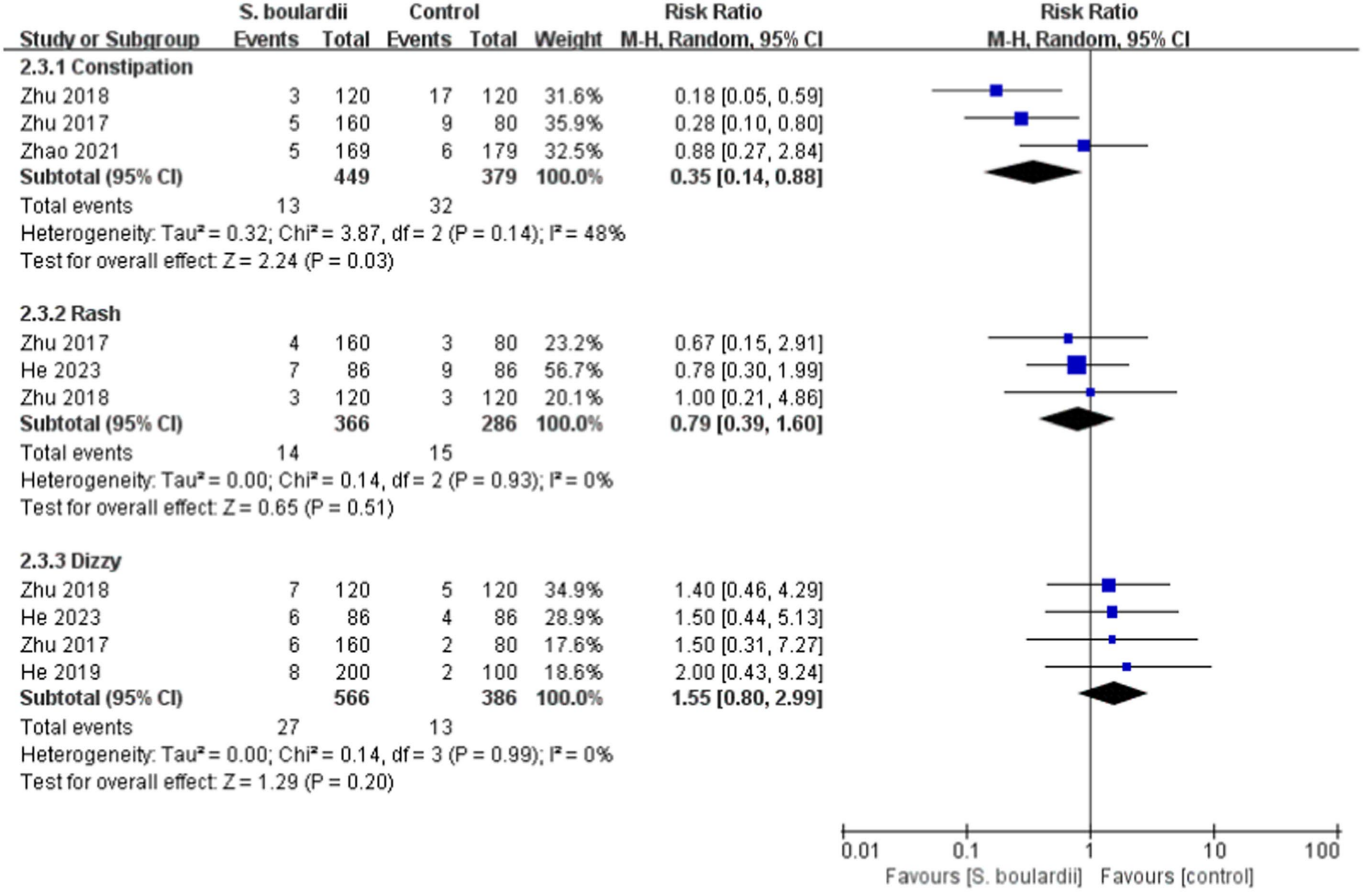
Forest plot of the specific adverse events (constipation, rash, and dizzy).

When it comes to specific adverse events, the results from the meta-analysis showed that those in the *S. boulardii* combined BQT group experienced a lower rate of diarrhea (*n* = 5 RCTs, 7.9% vs. 25.7%, RR = 0.29, 95% CI: 0.17–0.48, *p* < 0.00001), constipation (*n* = 3 RCTs, 2.9% vs. 8.4%, RR = 0.35, 95% CI: 0.14–0.88, *p* = 0.03) and abdominal distention (*n* = 4 RCTs, 4.9% vs. 12.7%, RR = 0.41, 95% CI: 0.23–0.72, *p* = 0.002) than the BQT group. Nonetheless, no significant difference was seen between the two groups in regards to nausea, vomiting, abdominal pain, rash and dizzy. The results of adverse events were presented in Figures 5, 6.

## 4 Discussion

### 4.1 Main findings and potential explanations

To our knowledge, this is the first time that a meta-analysis has been conducted to explore the effects of combining *S. boulardii* with BQT for the eradication of *H. pylori* compared to BQT alone. In this meta-analysis of six RCTs with 1,404 participants, we demonstrated that, when analyzed by ITT or PP, the eradication rate of the *S. boulardii*-supplemented BQT group was higher than that of the single BQT group. However, in PP analysis, there was no statistically significant difference between the two groups. It is worth noting that the 95% CI for both ITT and PP analyses overlapped with the invalid line, which could be due to the sample size being too small to draw statistically significant conclusions, or the high eradication rate of BQT, making it difficult to see the effects of adding *S. Boulardii*. In order to explore the effect of different durations of BQT regimen, different dosages and durations of *S. boulardii* on the overall results, we conducted subgroup analyses. The results showed that, although the eradication rate of *H. pylori* in the group supplemented with *S. boulardii* was higher than that in the single BQT group, except for the subgroup that was treated with *S. boulardii* for 14 days, there was no statistically significant difference between the two groups in the other subgroups. With such a small number of studies included in these subgroups, it may be difficult to draw statistically significant conclusions. Our meta-analysis also demonstrated that taking *S. boulardii* can reduce the occurrence of overall adverse events, diarrhea, abdominal distention, and constipation while eradicating *H. pylori*.

*Saccharomyces boulardii*, a fungal probiotic preparation, was originally isolated from tropical fruit peels. It is stable over a wide pH range, including acidic conditions and temperature levels, as well as during contact with bile salts and gastrointestinal enzymes (29). Due to its natural properties, the fungus is impervious to the antibiotic. Furthermore, the introduction of *S. boulardii* CNCM I-745 cannot generate antibiotic resistance since the exchange of antibiotic resistance genes with bacteria is improbable (30, 31). Evidence from current studies suggests that *S. boulardii* can successfully combat *H. pylori* infection both *in vitro* and *in vivo*. *S. boulardii* has the ability to directly inhibit *H. pylori* through the production of lactic acid, short-chain fatty acids, bacteritin, hydrogen peroxide, neuraminidase, and other substances (28). Furthermore, compared to other probiotic bacterial strains, *S. boulardii* has a much larger volume, resulting in a greater surface area and improved ability to adhere to pathogenic bacteria, thus impacting the colonization of *H. pylori* in the gastric mucosa (32). *S. boulardii* has neuraminidase activity that is specific to alpha (2–3)-linked sialic acid, and it acts by attaching itself to the adhesin of *H. pylori*, thereby preventing the adhesion of *H. pylori* in the duodenum (33). Moreover, *S. boulardii* can promote immunoprotection by triggering the secretion of sIgA and immunoglobulin in the gastrointestinal tract (34). Additionally, *S. boulardii* has an impact on the gut microbiota, thus decreasing gastrointestinal issues in patients (35), which leads to increased compliance and, as a result, a higher eradication rate of *H. pylori*.

### 4.2 Comparison with previous work

Previously, Yao et al. (36) conducted a meta-analysis of 10 RCTs and explored the effect of probiotic-supplemented BQT for the treatment of *H. pylori.* The results of the meta-analysis indicated that the eradication rate of the probiotic-supplemented BQT group was higher than that of the BQT group alone in both ITT (RR = 1.07, 95% CI: 1.02–1.11, *p* = 0.003) and PP analyses (RR = 1.04, 95% CI: 1.00–1.07, *p* = 0.03). In addition, probiotic supplementation was associated with a lower rate of side effects, diarrhea, and a bitter taste. Nevertheless, the meta-analysis included different probiotics, with only one study using *S. Boulardii*, which may lead to an inaccurate conclusion due to the strain specificity of probiotics and the fact that not all probiotics improve the *H. pylori* eradication rate or reduce the incidence of side effects (37). In comparison to the prior meta-analyses, our meta-analysis was more reliable due to the fact that it only focused on a particular probiotic strain (*S. Boulardii*) for consolidation.

### 4.3 Strengths and limitations

This meta-analysis has the major advantage of providing the latest and most comprehensive data on the impact of utilizing a single probiotic strain (*S. boulardii*) in combination with BQT to evaluate the eradication of *H. pylori* compared to BQT. In addition, we employed a rigorous systematic review methodology, employing a comprehensive search strategy, explicit inclusion and exclusion criteria, strict quality assessment, and strictly adhering to PRISMA statement for reporting, all of which ensured our results were transparent and reliable.

Despite this, this study still has certain limitations. First, out of all the studies we included, only one was a placebo-controlled double-blind trial, while the others did not include placebos. The blinding method and allocation were unclear, which could have an impact on our subjective outcome indicators (e.g., incidence of adverse events). Therefore, in the future, it is necessary to further conduct high-quality, placebo-controlled, double-blind trials to further verify these findings. Second, subgroup analyses only involve limited data, which can make it difficult to identify significant differences. Third, with the limited data available, it is difficult to ascertain the ideal dosage and duration of *S. boulardii* to achieve the desired results. Further optimization and confirmation is needed through further research. Fourth, this study did not include the classic BQT, currently recommended by international guidelines, that consists of PPI, salt of bismuth, tetracycline and metronidazole. Further exploration of this limitation is necessary in future research. Additionally, as there were less than 10 studies included, further publication bias testing was not conducted. Nevertheless, the potential for bias cannot be entirely dismissed. Finally, our meta-analysis focused on Asian populations, thus the results of this study can be applied only to the Asian population, and further research is required to determine if the findings can be extended to other populations.

## 5 Conclusion

Current evidence suggests that supplementation with *S. boulardii* in BQT may not have a major effect on the *H. pylori* eradication rate, but significantly reduces the incidence of overall adverse events, diarrhea, abdominal distention and constipation. Combining *S. Boulardii* with BQT can help alleviate symptoms, potentially improving patient adherence and offering a valuable treatment option.

## Data availability statement

The original contributions presented in the study are included in the article/Supplementary material, further inquiries can be directed to the corresponding author.

## Author contributions

YC: Writing – original draft, Conceptualization, Data curation, Formal analysis, Investigation, Methodology, Validation, Visualization. TT: Formal analysis, Methodology, Validation, Writing – original draft. YS: Data curation, Methodology, Validation, Writing – original draft. W-ZC: Conceptualization, Writing – review & editing.

## Footnotes

^1^ https://osf.io/n9z7c
