# Supplementary material for: The effect of supplementing with Saccharomyces boulardii on bismuth quadruple therapy for eradicating Helicobacter pylori: a systematic review and meta-analysis of randomized controlled trials
Source: Front Med (Lausanne). 2024 Apr 17;11:1344702. doi: 10.3389/fmed.2024.1344702 (PMC11061494; doi:10.3389/fmed.2024.1344702)
Supplement: Supplementary file 1 [file Data_Sheet_1.PDF]

Supplementary online materials

Table S1. **The details of search strategy from the other electric databases**

Figure S1. **Risk of bias graph**

Figure S2. **Risk of bias summary**

Table S1. The details of search strategy from the other electric databases

Databases	Search strings
**EMBASE**	#1 'helicobacter'/exp OR helicobacter:ab,ti<br>#2 'helicobacter pylori'/exp OR 'helicobacter pylori':ab,ti<br>#3 'H.pylori':ab,ti<br>#4 'campylobacter pylori':ab,ti<br>#5 #1 OR #2 OR #3 OR #4<br>#6 'saccharomyces boulardii'/exp OR 'saccharomyces boulardii':ab,ti<br>#7 'S boulardii':ab,ti<br>#8 'probiotics'/exp OR probiotics:ab,ti<br>#9 'probiotic'/exp OR probiotic:ab,ti<br>#10 #6 OR #7 OR #8 OR #9<br>#11 'bismuth'/exp OR 'bismuth':ab,ti<br>#12 #5 AND #10 AND #11
**Cochrane library**	#1 MeSH descriptor: [Helicobacter] explode all trees<br>#2 (helicobacter):ti,ab,kw<br>#3 MeSH descriptor: [Helicobacter pylori] explode all trees<br>#4 (Helicobacter pylori):ti,ab,kw<br>#5 (H.pylori):ti,ab,kw<br>#6 (campylobacter pylori):ti,ab,kw<br>#7 #1 OR #2 OR #3 OR #4 OR #5 OR #6<br>#8 (saccharomyces boulardii):ti,ab,kw<br>#9 (S boulardii):ti,ab,kw<br>#10 MeSH descriptor: [Probiotics] explode all trees<br>#11 (probiotics):ti,ab,kw<br>#12 (probiotic):ti,ab,kw<br>#13 #8 OR #9 OR #10 OR #11 OR #12<br>#14 MeSH descriptor:[bismuth] explode all trees

	#15 (bismuth):ti,ab,kw<br><br>#16 #14 OR #15<br><br>#17 #7 AND #13 AND #16
**Web of science**	TS=(helicobacter OR "helicobacter pylori" OR "H.pylori" OR "campylobacter pylori") AND TS=("saccharomyces boulardii" OR "S.boulardii" OR probiotics OR probiotic) AND TS=(bismuth)

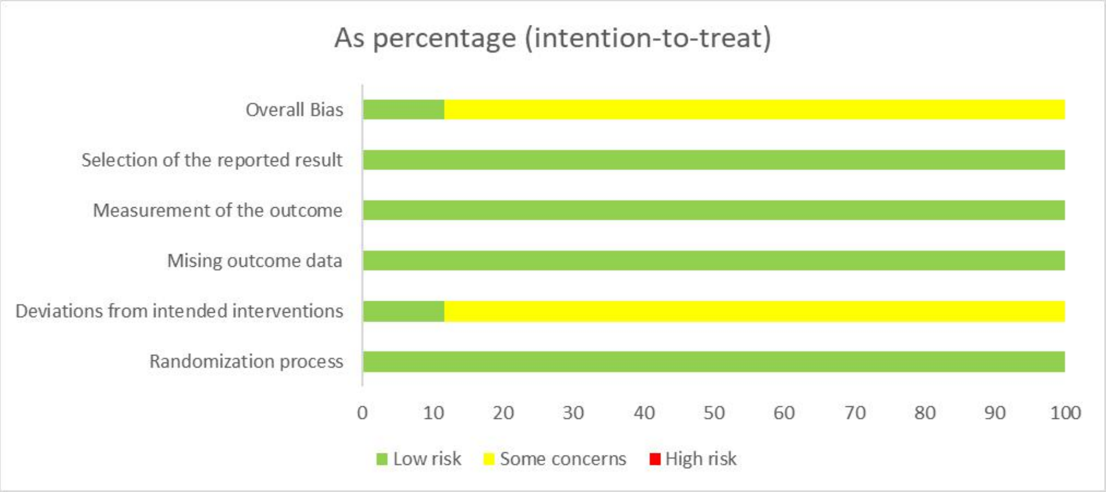

Figure S1. Risk of bias graph

Study ID	D1	D2	D3	D4	D5	Overall
Zhu 2017	+	!	+	+	+	!
Zhu 2018	+	!	+	+	+	!
He 2019	+	!	+	+	+	!
Zhao 2021	+	!	+	+	+	!
Naghibzadeh 2022	+	+	+	+	+	+
He 2023	+	!	+	+	+	!

+	Low risk
!	Some concerns

D1	Randomisation process
D2	Deviations from the intended interventions
D3	Missing outcome data
D4	Measurement of the outcome
D5	Selection of the reported result

Figure S2. Risk of bias summary
